# Correction: Parental perspectives on emergency health service use during the first wave of the COVID-19 pandemic in the United Kingdom: A qualitative study

**DOI:** 10.1371/journal.pone.0294795

**Published:** 2023-11-16

**Authors:** The PLOS ONE Staff

There are errors in the Funding section. The correct Funding statement is: MB is funded by, and JR and SB are members of the National Institute for Health and Care Research (NIHR) Applied Research Collaboration (ARC) North East and North Cumbria (NENC) (NIHR200173). The views expressed are those of the author(s) and not necessarily those of the NIHR or the Department of Health and Social Care.

The publisher apologizes for the error.
