# Supplementary material for: Parental perspectives on emergency health service use during the first wave of the COVID-19 pandemic in the United Kingdom: A qualitative study
Source: PLoS One. 2023 May 31;18(5):e0285375. doi: 10.1371/journal.pone.0285375 (PMC10231793; doi:10.1371/journal.pone.0285375)
Supplement: S1 File — (DOCX) [file pone.0285375.s001.docx]

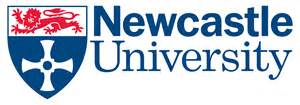


**Title: Use of children’s emergency services during the COVID-19 pandemic: a qualitative study of parents’ views and practice**

**Draft Topic guide**

1. **Since the coronavirus lockdown, have you had to make a decision about seeking healthcare for your child**

- What was the condition
- What was the decision
- Why was this decision made?
- If health services have been used, explore
  - Use of primary care services – GP, Walk-in centres
  - Use of 111 service
  - Use of emergency services
  - Experience of using services – e.g. modifications/precautions in place
  - Would they make the same decision again?

1. **(if not covered in Q1) In the current climate (i.e. told not to go out apart from work, shopping, exercise, health reasons. - update question to reflect current guidance) What would you do if you had a medical concern about your child?**

- Explore process e.g. self-manage, use of telephone services, pharmacy, GP and under what circumstances these would be used.
- Dependent on nature of condition? – how would they make this choice i.e. what criteria.
- To what extent would covid-19 feature in your decision to seek healthcare for your child?
- Do you feel that it would be safe to take your child to the ED?
- Generally worried about covid-19? Has this changed over time i.e. first heard about covid-19, when first cases appeared in UK, government guidelines, official lockdown etc.

2b. **(retrospective questions following potential changes in government guidelines) What would you have done if you had a medical concern about your child when the government guidance was only to leave home for exercise once per day, medical reasons and travelling to work?**

1. **Have you used children’s emergency services in the past? (IF NOT COVERED ABOVE)**

**–** What process did you go through e.g. call 111, contact/visit GP, straight to ED

- What type of reason?
- Decision making process?
- How did they travel?
- Advised to attend – e.g. 111, primary care
- Would they make the same decision today during the coronavirus pandemic? – why/why not? What would concerns be?

1. **In what ways has the coronavirus outbreak changed how you would use emergency health services for (i) yourself and (ii) your children?**

**4b. Are you aware of how other people you know may be using services differently?**

**-** in what way

**-** does this differ from your own view?

**4c. Do you have an understanding of how procedures in EDs may differ during the coronavirus pandemic to how they would have worked previously?**

- Does this affect your likelihood of visiting A&E

1. **Do you have enough information to make a decision about seeking healthcare for your child during the coronavirus lockdown?**

- What further information do you require?
- How should this information be presented/communicated?
- Who should provide this information?

1. **Is there anything else you would like to add?**

**Interviewer thanks the participant for taking part in the study and reminds them that their data will be held and used in confidence.**
