# Supplementary material for: Parental perspectives on emergency health service use during the first wave of the COVID-19 pandemic in the United Kingdom: A qualitative study
Source: PLoS One. 2023 May 31;18(5):e0285375. doi: 10.1371/journal.pone.0285375 (PMC10231793; doi:10.1371/journal.pone.0285375)
Supplement: Attachment — Submitted filename: Response to Reviewers.docx [file pone.0285375.s002.docx]

20^th^ February 2023

Dear Editor,

Thank you very much for the chance to amend our manuscript in line with the feedback received from the reviewers. We note that Reviewer #2 did not request changes but provided some general thoughts about our research but where amendments were requested by Reviewer #3 we have made changes.

Please find these changes detailed in the table below. We note that Reviewer #3 suggested that further review may not be necessary. We look forward to hearing from you.

Yours sincerely,

Matt Breckons

| Comment | Our response |
| --- | --- |
| **Reviewer #2** | |
| In summary, parents of children were recruited for interviews via social media and posters to discuss their decision making around accessing emergency healthcare services during the early COVID-19 pandemic. A skewed population was recruited; over 2/3 of participants had accessed healthcare services during the pandemic and the majority originate from areas of low deprivation.  Three themes were identified:  1) Making sense of risk. Whilst this is a key part of the paper, the findings mainly relate to the risk that interviewees felt at the time of accessing healthcare settings, rather than the anticipated risks which influenced their decision making around accessing healthcare settings.  2) Interpreting information on emergency healthcare services. This is a really key part of the paper. It is unfortunate that the thematic questioning did not include the use of online resources - NHS, RCPCH etc all released early guidance and understanding whether the use of these resources influenced decision making for families would be an important message to draw out for any future pandemics  3) Making the right decision. There appears to be a well-rounded description of view points within this theme. | Thank you for these comments. Your thoughts on parents views on risk are interesting; we anticipated that people would talk about perceived risk of attending healthcare settings but as you refer to, the fact that 2/3 had accessed healthcare services meant that perhaps a broader discussion of risk took place.  With regard to interpreting information on healthcare services. We agree that this is a key part of the paper. While we did not specifically mention these sources of information, it is interesting that these were not specifically mentioned; it was the case that participants discussed sources of information they used which included online information although we agree that a more ‘zoomed in’ discussion of information sources would be interesting.  Your comments re: skewed sample are interesting and does raise the question of how a less skewed population might be accessed – we will certainly take this learning forward for future work. |
| **Reviewer #3** | |
| Could you please capitalise COVID-19 and ensure this is used throughout. | Thank you, we have made this change throughout. |
| You may wish to explain that social distancing is a bit of a misnomer, when in fact the reality was physical distancing and we were encouraged to socially network. | Thank you for this comment. We appreciate the distinction being made more recently. We have considered how we have used this term and agree that ‘physical distancing’ is what we’re referring to in most cases and have revised accordingly. |
| A personal point of pedantry - line 54 of the revised introduction, can you re-phrase the beginning of the sentence so it doesn't refer to 'A 2020 paper' - when there are no written references with this style it adds a mystery to the tone which is confusing - perhaps name the authors? | Thank you we have amended this accordingly. |
| Interview schedule not topic guide, the latter being reserved for unstructured interviews and focus groups - change throughout. | Thank you we have amended. |
| You state you recruited on IMD Decile, but you report IMD grouped as Quintiles - perhaps explain why? | Thank you this is a great point. We have added a footnote to the table to clarify. |
| Could a note be put in as to why some IMD data is missing? | We have added a note to clarify that IMD could not be calculated where an incomplete postcode was provided. |
| Lines 183 - 197 in revised results section - is it sheer coincidence that the participants in Interview 2 and Interview 15 have children of the exact same age? At first I thought this was two parents of the same children, and then I noticed that they were in different IMD groups, so I perhaps mistakenly assumed they were separated parents of the same children. I think some clarification might be needed here if, in fact, these two participants bear no relationship to one another. | Yes! We checked meticulously prior to submission but have just returned to these data to check. We appreciate this is coincidence in a small sample and, as such, have added clarification on the two occasions where we refer to Interview 15 – mentioning that there is no link to Interview 2. |
| Where you are redacting names in the results could you utilise the accepted '' rather than *Name of Son* - change throughout. | We have changed throughout. |
| Is a bit long for my liking, but I can see a lot of it comes from the edits you have had to make for the previous reviewer | Thank you. We agree with this comment. |
